# Regional bedrock geochemistry associated with podoconiosis evaluated by multivariate analysis

**DOI:** 10.1007/s10653-018-0158-0

**Published:** 2018-09-05

**Authors:** Jamey N. Cooper, Allen M. Cooper, Benjamin L. Clausen, Kevin E. Nick

**Affiliations:** 1 Department of Earth and Biological Sciences, Loma Linda University, Loma Linda, CA 92350 USA; 2 Geoscience Research Institute, Loma Linda, CA 92350 USA

**Keywords:** Podoconiosis, Rift volcanics, Principal component analysis, Multivariate analysis, Geochemistry

## Abstract

Podoconiosis is a disease whose etiology remains murky. Currently, the disease is attributed to particles that are believed to move through the skin and into the lymphatic system causing swelling of the lower legs. Identity of these particles or their composition remains unclear, though the presence of silicon and/or aluminum is often noted and frequently cited as causal agents. We applied multivariate analyses to the bedrock compositions of a large set of cases from an online database in an effort to identify underlying patterns or combinations of relative element abundances associated with podoconiosis-endemic regions. Using a combination of principal component analysis, discriminant function analysis, and ANOVA, we analyzed ten oxides from five regions on the African continent known to be associated with podoconiosis. The Hawaiian Islands were included as a control group since they are not known to have cases of podoconiosis despite similarity in geology and agricultural practices. Our analyses suggest that a unique alkaline- and silicon-rich geochemistry underlies regions associated with podoconiosis. Our results also imply that minerals enriched in incompatible elements, such as Ca, K, Mg, and Na, may be stronger predictors of the presence of the disease than either silicon or aluminum.

**Electronic supplementary material:**

The online version of this article (10.1007/s10653-018-0158-0) contains supplementary material, which is available to authorized users.

## Introduction

One of the greatest challenges in many areas of research is uncovering meaningful patterns and relationships within large, complex data sets. Multivariate statistical analyses are valuable tools for probing such data sets, allowing investigators to, among other things, identify underlying structure present in a set of variables, as with principal component analysis (PCA), and classify subjects into groups, as with discriminant function analysis (DFA). The use of these methods is not new to geology. Bisani et al. (1983) analyzed 11 major and 13 trace elements related to igneous rock composition from five islands in the Aeolian Archipelago using PCA and found that the islands could be distinguished from one another based on four components. They also found the results supported process models of rock formation. Similarly, Grunsky et al. (1992) used PCA to reduce the number of dimensions required to identify a particular magma group. Le Maitre (1976) used DFA to classify rock samples into either basalts, andesites, dacites, or rhyolites using their major oxide composition. He believed this method could be a satisfactory way to relate the chemistry of a rock to its mineralogical classification in a more quantitative way. Pearce (1976) successfully employed DFA to classify various types of basalts. A very convincing use of multivariate methods for geological interpretation was Ragland et al. (1997) methodical use of both PCA and DFA to analyze the Martinsville igneous complex. Comparing the results of multivariate analyses to more traditional geological approaches, they found that not only did the multivariate approaches group and classify the data the same as more traditional approaches but they also retained and portrayed the petrogenic significance of several mineralogical groupings such as mixing and control line populations. We employed this approach to look for distinguishing characteristics of regions known to have podoconiosis.

Multivariate analyses are especially valuable for analyzing large data sets such as are available from GEOROC (http://georoc.mpch-mainz.gwdg.de/georoc/), NAVDAT (www.navdat.org), National Geochemical Database (https://mrdata.usgs.gov/ngdb/rock/), or EarthChem (http://ecp.iedadata.org/). Access to these large bodies of data allows researchers to look for underlying trends that might not be apparent in smaller data sets collected by single individuals. The global nature of these data provides the potential for comparisons not only regionally, but across continents. In addition, these databases provide access to data from regions to which a researcher may not be able to travel due to budget, time, or political constraints. Of course, the real benefits to using large data sets are the sheer number of samples that can be analyzed. Patterns that would have been hidden when analyzing dozens of samples become visible when thousands of data points are included.

Podoconiosis is a little-known disease that causes swelling and disfiguration of the lower legs. Podoconiosis occurs in several developing countries where shoes are not worn on a regular basis. Worldwide, podoconiosis affects about four million people in at least 34 countries, with major areas of incidence in Africa, South America, and the Indian subcontinent (Loewenthal 1934; Clark 1948; Jordan et al. 1956; Cohen 1960; Price 1975, 1990; Price and Henderson 1981; Price and Bailey 1984; Corachan et al. 1988; Ruiz et al. 1994). Ethiopia has the unfortunate distinction of being the country most highly affected, with recent geostatistical modeling estimates of 1,539,963 affected individuals in 2015 (Deribe et al. 2017). The etiology of podoconiosis is still unclear, but as far back as the 1800s a correlation between podoconiosis and the environment led to many theories that the soil and a subcomponent were causative agents. The association between volcanic soils and agricultural communities in which footwear is often a luxury strengthened the mineral-related hypothesis. Through daily work in agricultural fields, individuals’ bare feet are in constant contact with the soil, and thus with the particles believed to induce podoconiosis (Price and Bailey 1984). However, epidemiological work over the years has shown the disease to be highly localized (Oomen 1969; Price 1974b; Deribe et al. 2013, 2017). This suggests enrichment of a particular component either due to either a specific bedrock geochemistry underlying endemic locations and/or unique weathering patterns during soil formation.

During the 1970s and 1980s, the physician Ernest Price believed he had narrowed down the cause to a “tropical red soil,” classified as ultisol or ferrosol, formed through the weathering of alkalic volcanic rocks (Price 1974b, 1976). Price and his colleagues sought to determine whether this association between soil and disease was one that could be demonstrated within the body. They studied the lymph nodes of patients living in affected and unaffected regions and found particles containing silicon, aluminum, and iron inside the nodes of both groups of patients (Heather and Price 1972). Having established the presence of particles within the lymph system, the authors tried to determine whether a difference in the quantity of particles existed between diseased patients and those not exhibiting symptoms. Price and Henderson (1978) suggested that more birefringent particles, interpreted as inorganic particles, were found in diseased patients, but were unable to establish whether elephantiasic nodes contained more total particles than the non-elephantiasic. In a later paper, Price and Henderson (1979) did, however, report a difference in the numerical distribution of particles exhibiting various Al/Si ratios between diseased patients and those not exhibiting symptoms. Further work also revealed higher numbers of submicron-sized clay particles, specifically kaolinite, in the disease-associated soils (Price and Bailey 1984). A summary model proposes that weathering of source rock leads to the formation of clay-rich soils, and that fine, clay-sized particles are the source of the irritating nature of the soil (Price and Henderson 1979; Price et al. 1981; Price and Bailey 1984). More recent work by Molla et al. (2014) reports no significant association with particle size, but did find smectite, quartz, and mica to display positive correlations. Le Blond et al. (2017) report a higher volume % of fine particles in endemic soils as well as higher amounts of Al_2_O_3_, Cr, kaolinite, mica, Ni, quartz, SiO_2_, Y, and Zr.

Several factors play a role in the formation of soils including parent material (bedrock), climate, organisms, relief, and time (White 2013). This process becomes infinitely more complex as each of these factors may change over time leading to different factors playing the dominant role in pedogenesis. In an effort to more fully understand the origins of podoconiosis, each of these factors must be examined individually. Previous podoconiosis research has investigated both climate, identifying modern cases to be limited to tropical environments, and relief, correlating increased incidence of the disease with higher elevations (Price 1976; Price and Bailey 1984; Molla et al. 2014; Deribe et al. 2015). In this paper, we focus on the parent material from which soils are derived. It has been noted by geologists that the orogenesis of the East African Rift contains individual igneous provinces that contain petrological and geochemical signatures from a variety of magma types including tholeiitic, transitional, alkaline, and ultra-alkaline (Kampunzu and Mohr 1991; Kampunzu and Popoff 1991). Heterogeneity in soil type from Ethiopian soil maps suggests that the soils are locally derived (Schlüter 2006). The data from Le Blond et al. (2017), in particular, provide an excellent point of comparison between the bedrock data and soil data. By using these two in conjunction, we can begin to piece together a story for what geological pathway might lead to a soil composition which plays a role in the onset of podoconiosis. To investigate bedrock chemical compositional factors related to podoconiosis, we compiled a large data set from five regions known to have (currently or historically) published cases of podoconiosis: Cameroon Line, Cape Verde Islands, Mid-African Rift System, East African Rift System, and the Red Sea Rift. Geological histories suggest an influence from hot spot activity for all regions, and from rifting for all except the Cape Verde Islands (Fitton 1987; Déruelle et al. 1991; Kampunzu and Mohr 1991; Kampunzu and Popoff 1991; Anderson and Schramm 2005; Ramalho 2011). The Hawaiian Islands were used as an out-group due to the lack of podoconiosis despite similarity to the African regions both in their geological origin (as volcanic centers associated with rifting and plume mechanics) and the manual agricultural techniques historically used there (Kirch 1997; Kirch and Zimmerer 2011). The goal of this study is to see whether a statistical comparison of the geochemistry from a large data set among these regions would identify a specific composition or element that suggests petrological processes unique to regions associated with podoconiosis.

## General methods

### Data source, screening, and transformation

Weight percentage data for 13 oxides (SiO_2_, Al_2_O_3_, MgO, Fe_2_O_3_, TiO_2_, MnO, CaO, Na_2_O, K_2_O, P_2_O_5_, B_2_O_3_, Cr_2_O_3_, and NiO) were downloaded September 2012 from the Geochemistry of Rocks of the Oceans and Continents (GEOROC) Web site (http://georoc.mpch-mainz.gwdg.de/georoc/) for five regions hosting published cases of podoconiosis (Cameroon Line, Cape Verde Islands, Mid-African Rift System, East African Rift System, Red Sea Rift) and one control region (Hawaiian Islands) giving a starting sample size of *n* = 14,527 cases. For each case, weight percentages for the oxides were summed and, following Kovacs et al. (2001), those cases whose total percentage fell between 98 and 102% (*n* = 12,013) were retained for further analysis. Due to low sample numbers, the oxides B_2_O_3_, Cr_2_O_3_, and NiO were omitted from analyses.

Zeroes in the original data were treated as rounded zeros (below detection values) and replaced using additive zero replacement (Aitchison 1986). All weight percentages were centered log-ratio (clr) transformed prior to statistical analysis (Grunsky 2010; Pawlowsky-Glahn and Egozcue 2012), where clr = ln(oxide weight%/geometric mean of composition). Using data from the remaining ten oxides, we identified multivariate outliers (*n* = 573) using Mahalanobis distance (Mertler and Vannatta 2005) and excluded these from analyses. Some cases (*n* = 887) were missing values for one or more of the ten oxides. For descriptive statistics, and AFM (Alkali [Na + K], Fe, Mg) and TAS (total alkali-silica) plots, pairwise deletion was employed resulting in a total *n* = 11,440, and minimum and maximum pairwise sample sizes for oxide by region of *n *= 36 (MnO, Cameroon Line) and *n* = 8319 (SiO_2_ and others, Hawaiian Islands), respectively (see Online Resource 1). A map of these locations is provided as Online Resource 2. Listwise deletion was employed for all other analyses giving a total (all regions included) *n* = 10,553. Insufficient cases remained from the Cameroon Line when listwise deletion was employed; therefore, data from this region were only included in descriptive statistics, and AFM and TAS plots. Thus, the final data set for PCA, DFA, and analysis of variance (ANOVA) (derived by listwise deletion) included five regions (Cape Verde Islands [*n* = 718], Mid-African Rift [*n* = 328], East African Rift System [*n* = 1926], Red Sea Rift [*n* = 79], Hawaiian Islands [*n* = 7502]) and ten oxides (SiO_2_, Al_2_O_3_, MgO, Fe_2_O_3_, TiO_2_, MnO, CaO, Na_2_O, K_2_O, and P_2_O_5_).

### Statistical analyses

All statistical tests were conducted using SPSS 23.0 for Windows (Statistical Package for the Social Sciences, Inc., Chicago, Illinois, USA) with *α* = 0.05. Following Nakagawa (2004), we chose not to adjust *α* for multiple tests. Despite centered log-ratio transformation, data did not strictly meet parametric assumptions. Unless indicated otherwise, measures of central tendency presented are mean ± 1S.E. Individual models are specified in Results section. We further computed effect sizes, which are independent of sample size (in contrast to statistical significance) and more readily compared among different data sets and different studies (Hojat and Xu 2004; Nakagawa and Cuthill 2007). For pairwise comparisons, we relied on Cohen’s *d* using pooled standard deviation (Hojat and Xu 2004), for which values of ~ 0.2, ~ 0.5, ≥ 0.8 are generally considered small, moderate, and large, respectively (Cohen 1988). For DFA and ANOVA, we computed multivariate eta-squared (*η*^2^) and *η*^2^, respectively, with values of ~ 0.01, ~ 0.06, and ≥ 0.14 loosely regarded as small, moderate, and large, respectively (Cohen 1988). Cohen’s d provides a standardized unit of difference, whereas η^2^ indicates the approximate proportion of variance explained.

## Results

### Descriptive statistics, and AFM, and TAS

**Fig. 1:**
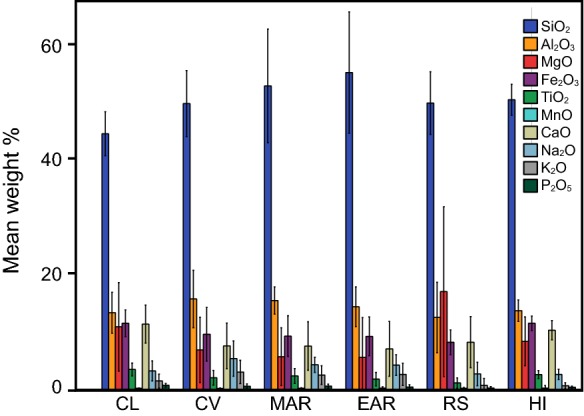
Mean ± 1 SD weight percentage (untransformed) of 10 oxides for the six regions (CL = Cameroon Line, CV = Cape Verde Islands, MAR = Mid-African Rift, EAR = East African Rift, RS = Red Sea Rift, HI = Hawaiian Islands) analyzed (total *n* = 11,440; min. and max. pairwise sample sizes for oxide by region of *n* = 36 and *n* = 8319, respectively)

The mean and standard deviation of untransformed weight percentages of SiO_2_, Al_2_O_3_, MgO, Fe_2_O_3total_, TiO_2_, MnO, CaO, Na_2_O, K_2_O, and P_2_O_5_ by region are reported in Fig. 1 (a table with these values and additional descriptive statistics can be found in Online Resource 2). Mean oxide percentages vary considerably for a given region, and there is substantial variation across regions for a given oxide. SiO_2_ shows the largest region means of any oxide, ranging between 44 and 54%. Al_2_O_3_ has the next highest means with values of 13–17%. Fe_2_O_3total_, CaO, and MgO have similar region means ranging between 5 and 11%, and the remaining oxides have region means of less than 3%. The coefficient of variation (CoV; see Online Resource 2, data table) ranges quite dramatically among regions for most of the oxides.

**Fig. 2:**
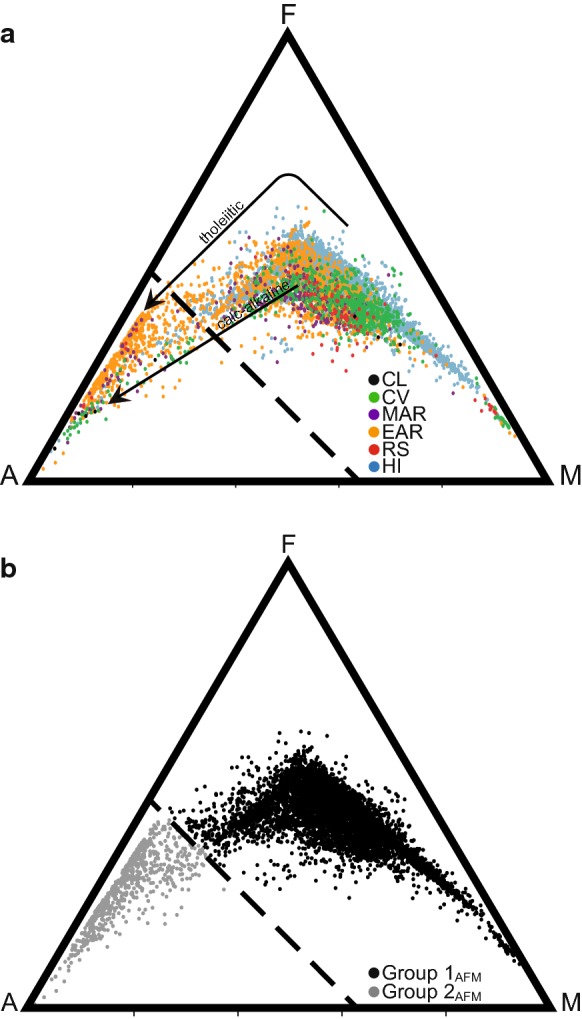
AFM (Alkali [Na+K], Fe, Mg) diagram of untransformed data (*n* = 11,440) color coded to show **a** six regions (CL = Cameroon Line, CV = Cape Verde Islands, MAR = Mid-African Rift, EAR = East African Rift, RS = Red Sea Rift, HI = Hawaiian Islands) and **b** Group 1_AFM_ and Group 2_AFM_ created by clustering of data and defined by visual inspection according to the line y = (−75/63)(MgO%+Fe_2_O_3_total)/2) + 75

**Fig. 3:**
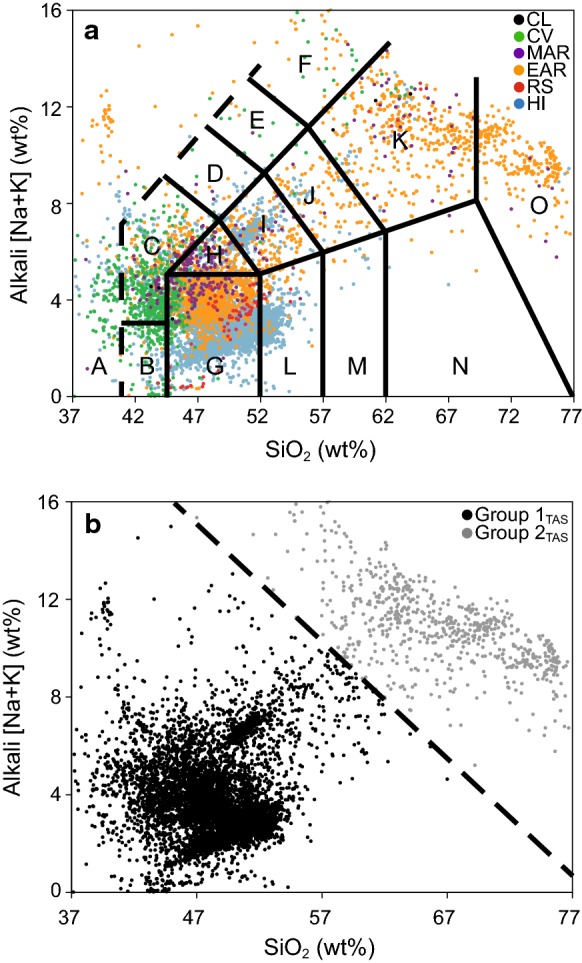
TAS (total alkali silica) diagram of untransformed data (*n* = 11,440) color coded to show **a** six regions (CL = Cameroon Line, CV = Cape Verde Islands, MAR = Mid-African Rift, EAR = East African Rift, RS = Red Sea Rift, HI = Hawaiian Islands). Names of equivalent rock types: (A)-Foldite, (B)-Picrobasalt, (C)-Tephrite/Basanite, (D)-Phonotephrite, (E)-Tephri-phonolite, (F)-Phonolite, (G)-Basalt, (H)-Trachybasalt, (I)-Basaltic trachyandesite, (J)-Trachy-andesite, (K)-Trachyte/Trachydacite, (L)-Basaltic andesite, (M)-Andesite, (N)-Dacite, (O)-Rhyolite (Le Maitre et al. 2002). **b** The same diagram color coded to show Group 1_TAS_ and Group 2_TAS_ created by clustering of data and defined by visual inspection according to the line y = ((−19.5/42)(SiO_2_ wt%)) + 36.7

Plotting an AFM diagram (Fig. 2a) using untransformed data for the six regions demonstrates both a tholeiitic differentiation trend and a calc-alkaline differentiation trend. For each of the African regions, data points on the AFM diagram separate into two visually distinct clusters, a phenomenon not observed for Hawaiian data points. Using visual inspection, we created a line, defined by the equation $y = \left( { - \frac{75}{63}} \right)\left( {\left( {{\text{MgO}}\% + \frac{{{\text{Fe}}_{2} {\text{O}}_{3} }}{2}} \right) + 75} \right)$, that separates the two clusters. It is of note that cases from all regions studied can be found in both groups. However, the majority of all cases, and almost all the Hawaiian Island cases, fall into one cluster, while the second cluster is comprised of a majority of East African Rift cases. The first group (located to the right of the dashed line on the AFM diagram) is high in MgO, intermediate to high in Fe_2_O_3total_, and very low in Na_2_O and K_2_O. The second group (located to the left of the dashed line on the AFM diagram) is very low in both MgO and Fe_2_O_3total_ and very high in Na_2_O and K_2_O. We coded the separated data points into a dichotomous grouping variable (Groups 1_AFM_ and 2 _AFM_, Fig. 2b). A TAS diagram shows a similar clustering of data points into two groups (Fig. 3a) that we separated along the line, $y = \left( { - \frac{19.5}{42}} \right)({\text{SiO}}_{2} \% ) + 36.7$, as indicated (dashed line) in Fig. 3b. As before, we coded the separated data points into a dichotomous grouping variable (Groups 1_TAS_ and 2 _TAS_, Fig. 3b). Note that Group 2 _TAS_, like Group 2 _AFM_, falls into the high alkaline category comprised of phonolite, trachyte, dacite, and rhyolite. Group 1_TAS_ is highly basaltic in its origin.

### Analysis of oxides by region

#### Principal component analysis

**Fig. 4:**
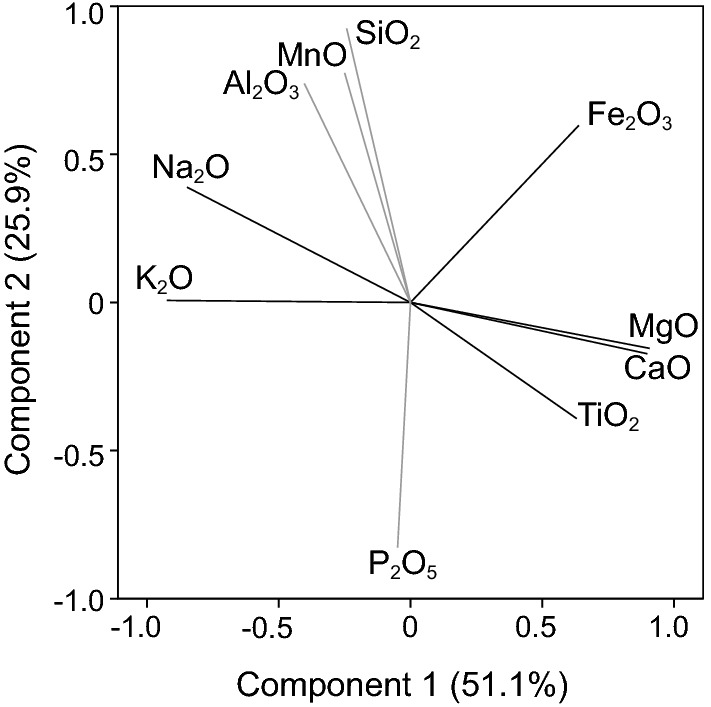
Factor loadings for varimax rotated components from principal component analysis of 10 oxide variables (centered log-ratio transformed, *n* = 10,553)

We employed PCA to investigate what latent structure is present in the set of clr-transformed weight percentages of ten oxides (SiO_2_, Al_2_O_3_, MgO, Fe_2_O_3total_, TiO_2_, MnO, CaO, Na_2_O, K_2_O, and P_2_O_5_; total *n* = 10,553) that might relate to regions known to have podoconiosis. Principal component analysis was conducted using a varimax rotation. Eigenvalue and variance criteria (Mertler and Vannatta 2005) indicate a two-component solution, with components 1 and 2 accounting for 51.1 and 25.9% of the total variance in the original variables, respectively. Figure 4 presents the loadings for each component. The highest loading oxides on component 1 are K_2_O, MgO, and CaO with 92.5, 90.8, and 89.9% of their variability, respectively, explained by the two-component solution. Oxides with highest loadings on component 2 are SiO_2_ and P_2_O_5_ with 91.5 and 68.7% of their variability, respectively, explained by the model.

**Fig. 5:**
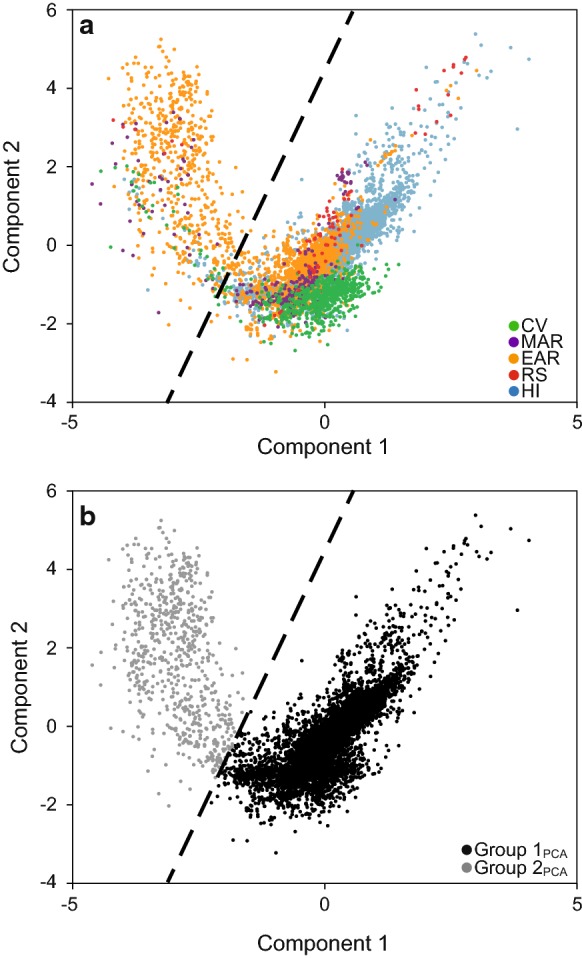
Principal component scores on components 1 and 2 (centered log-ratio transformed data [*n* = 10,553]) color coded to show **a** five regions and **b** Group 1_PCA_ and Group 2_PCA_ created by clustering of data and defined by visual inspection according to the line y = ((−5.5/2)(Comp1)) + 45. Note that group separation is greatest along component 1 (associated with incompatible K and Na and compatible Mg, Ca) and minimal along component 2 (associated with Si)

When cases are plotted based on component scores (Fig. 5a), two clusters of data points become visible (reminiscent of those seen in the AFM diagram (Fig. 2a) and the TAS diagram (Fig. 3a)). Those two clusters, named Group 1_PCA_ and Group 2_PCA_, were separated based on visual inspection, along the line defined by the equation $y = \left( { - \frac{5.5}{2}} \right)({\text{component}}\,1)$, (Fig. 5b). An independent samples *t* test determines that scores on principal component 1 are larger for Group 1_PCA_ (0.19 ± 0.01) than for Group 2_PCA_ (− 2.69 ± 0.02; *t*(853.66) = 124.01, *p* < 0.001, Cohen’s *d* = 4.14). In contrast, scores on principal component 2 are larger for Group 2_PCA_ (1.98 ± 0.06) than for Group 1_PCA_ (− 0.14 ± 0.01; *t*(727.14) = − 35.38, *p* < 0.001, Cohen’s *d* = 2.5). Examination of component scores (Fig. 5b) shows that component 1 provides the largest separation between Group 1_PCA_ (characterized by larger relative amounts of MgO and CaO) and Group 2_PCA_ (characterized by larger relative amounts of K_2_O and Na_2_O). By comparison, component 2 provides only minor separation between groups (with Group 1_PCA_ characterized by smaller relative amounts of SiO_2_ and Al_2_O_3_). Effect sizes reveal that component 1 contributes more to the separation of Group 1_PCA_ and Group 2_PCA_ than does component 2.

**Fig. 6:**
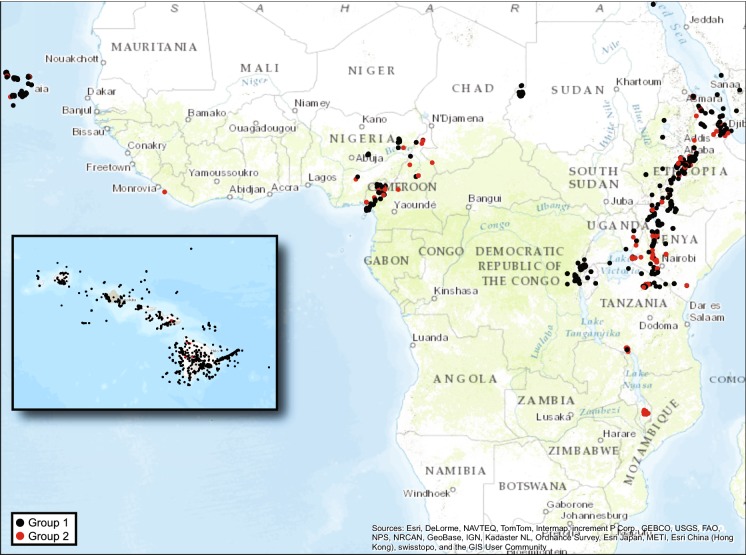
Locations of individual cases (*n* = 10,553) used in this study color coded into Group 1_PCA_ (*n* = 9849) and Group 2_PCA_ (*n* = 704). Considerable overplotting occurs at this scale

The efficacy of visual separation of Hawaiian and East African Rift cases using the line in Fig. 5b was confirmed by comparing the proportions of Groups 1_PCA_ and 2_PCA_ comprised of Hawaiian and East African Rift cases (Table 1); while 76% of Group 1_PCA_ cases are from the Hawaiian Islands, only 7% of Group 2 cases are Hawaiian. In contrast, 80% of Group 2_PCA_ cases are from the East African Rift. Comparing the mean weight percentages of each oxide for Groups 1_PCA_ and 2_PCA_ (Table 2) reveals relatively large differences between the groups, as measured by Cohen’s *d*, for almost every oxide. Ranking oxides by absolute value of Cohen’s *d* shows, not unexpectedly, a pattern similar to that seen when ranking oxides by magnitude of loadings from PCA, with larger standardized differences between Group 1_PCA_ and 2_PCA_ means observed for Na_2_O, CaO, MgO, K_2_O, and TiO_2_, the oxides loading predominantly on component 1. Smaller effect sizes are observed for SiO_2_, Al_2_O_3_, MnO, and P_2_O_5_, the oxides loading predominantly on component 2. Cases comprising Group 1_PCA_ and Group 2_PCA_ were mapped to look for geographic patterns in the data (Fig. 6), but no obvious patterns are visible.

**Table 1:** Number of cases from each region comprising PCA Groups 1 and 2, and the percentage of PCA Groups 1 and 2 cases by region (CV = Cape Verde Islands, MAR = Mid-African Rift, EAR = East African Rift, RS = Red Sea Rift, HI = Hawaiian Islands)

Location	PCA Group 1 *n*	% of PCA Group 1 cases	PCA Group 2 *n*	% of PCA Group 2 cases
CV	683	7	35	5
MAR	279	3	49	7
EAR	1364	14	562	80
RS	73	< 1	6	< 1
HI	7450	76	52	7
Total	9849	100	704	100

Note that 76% of Group 1 cases are from the Hawaiian Islands while 80% of Group 2 cases are from the East Africa Rift

**Table 2:** Mean ± 1 SD weight percentage (untransformed) of ten oxides for PCA Groups 1 (*n* = 9849) and 2 (*n* = 704). Cohen’s *d* for the difference in means (calculated using centered log-ratio transformed data) between Group 1 and Group 2 for each oxide is also provided

Oxide	PCA Group 1		PCA Group 2		Cohen’s *d*
	Mean	SD	Mean	SD	
Na_2_O	2.74	0.98	6.00	1.40	− 5.53
CaO	10.32	1.80	2.22	2.54	5.31
MgO	8.29	4.38	1.08	2.73	4.75
K_2_O	0.81	0.77	4.30	1.23	− 4.22
TiO_2_	2.62	0.75	0.80	0.71	3.84
SiO_2_	49.36	3.19	64.33	7.36	− 3.19
Al_2_O_3_	13.85	2.11	14.78	3.09	− 3.05
MnO	0.18	0.04	0.22	0.09	− 2.75
P_2_O_5_	0.40	0.28	0.20	0.25	1.58
Fe_2_O_3_	11.42	1.34	6.07	2.27	1.02

Note the higher mean values of K_2_O, Na_2_O, and SiO_2_ for PCA Group 2

**Fig. 7:**
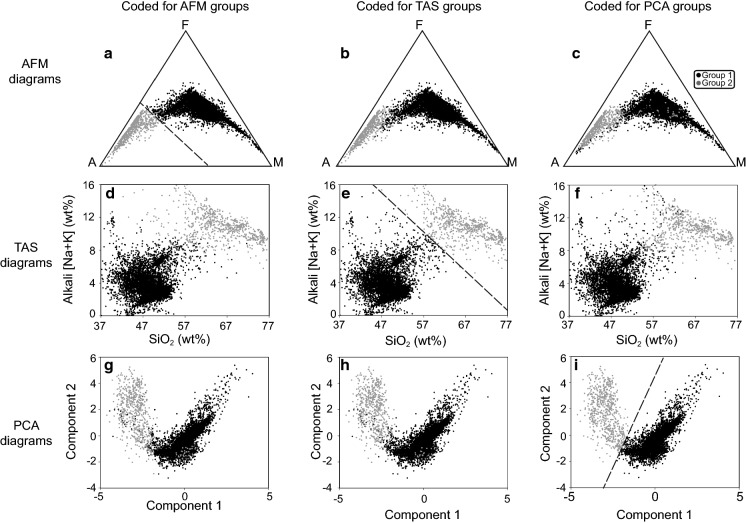
AFM, TAS and PCA diagrams coded to visualize similarity in classification of cases into groups. **a** AFM original, **b** AFM with cases colored according to TAS groups, **c** AFM with cases colored according to PCA groups, **d** TAS with cases colored according to AFM groups, **e** TAS original, **f** TAS with cases colored according to PCA groups, **g** PCA with cases colored according to AFM groups, **h** PCA with cases colored according to TAS groups, **i** PCA original

**Fig. 8:**
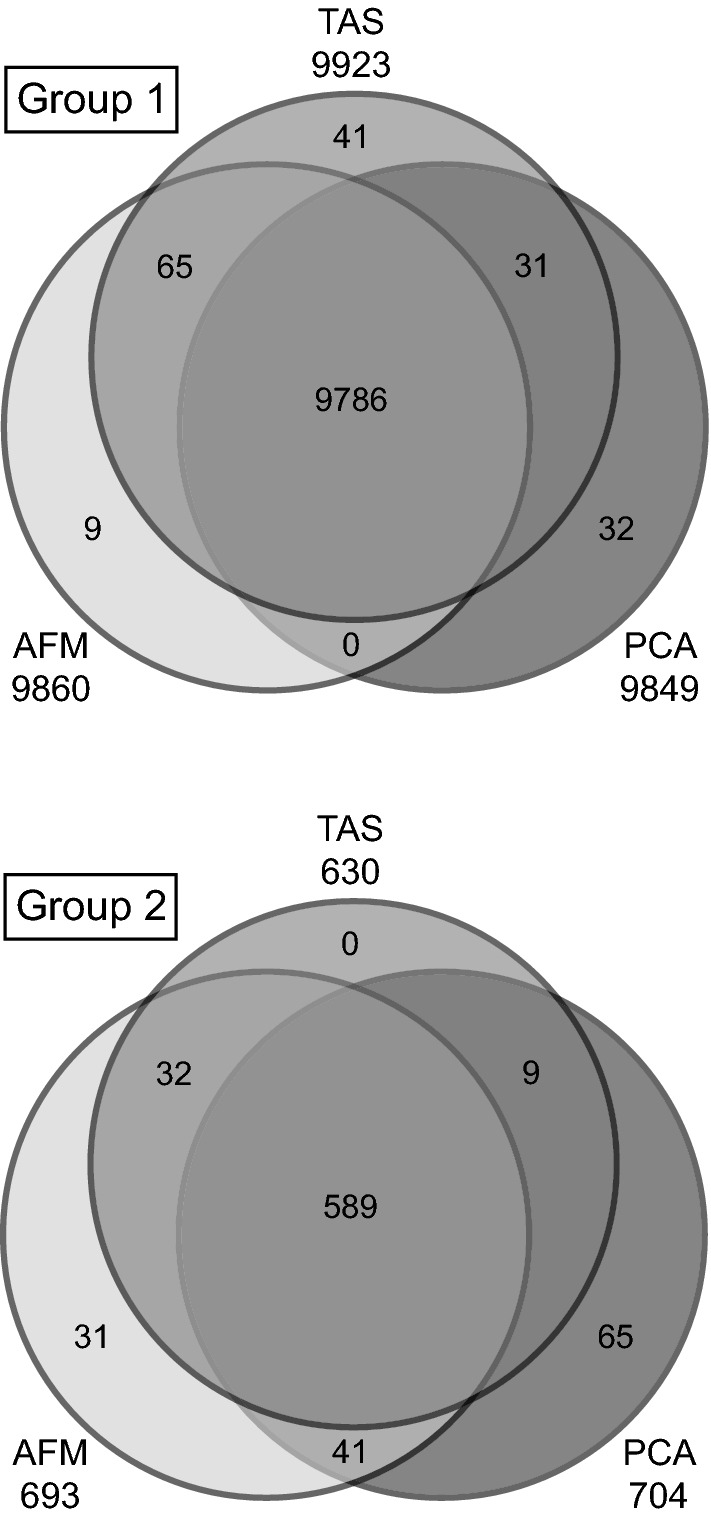
Number of cases classified similarly into Group 1 and Group 2 using the approaches of AFM, TAS, and PCA. Venn diagrams only include the *n* = 10,553 cases common to all three approaches. Areas of overlap are not to scale

Close correspondence of the cases between AFM, TAS, and PCA groups was suspected due to the variables in common among the three approaches (i.e., oxides utilized in AFM and TAS plots are subsets of those used for PCA, and AFM and TAS have alkali oxides in common). Indeed, close correspondence is confirmed upon examination of the overlap in case classification (Fig. 7); 10,375 (98.3%) of the 10,553 cases were classified similarly by the three approaches. The similarity among corresponding groups is visually apparent upon comparing AFM, TAS, and PCA diagrams with groups defined by means of alternative plotting methods (Fig. 8).

#### Discriminant function analysis

A direct discriminant function analysis was performed using the clr-transformed weight percentages of ten oxides (SiO_2_, Al_2_O_3_, MgO, Fe_2_O_3total_, TiO_2_, MnO, CaO, Na_2_O, K_2_O, and P_2_O_5_) as predictors of a case’s geographic region of origin (Cape Verde Islands, Mid-African Rift, East African Rift, Red Sea Rift, and Hawaiian Islands; *n* = 10,553). Prior probabilities were computed from group sizes (Mertler and Vannatta 2005), and cases were classified based on leave-one-out classification, a jackknifing procedure that classifies each case by the functions derived from all cases other than the omitted case.

Four discriminant functions were calculated with a combined *Λ* = 0.41, *χ*^2^(40, *n *= 10,553) = 9450.38, *p* < 0.001, multivariate *η*^2^ = 0.20, indicating that region accounts for 20% of model variance. Table 3 presents structure matrix correlations and standardized function coefficients. All of the predictors have their highest proportion of variance extracted by either functions 1 or 2 (Table 3 structure matrix). Function 1 has 46% of its variance explained by region and accounts for 73% of the model’s between-groups variance. Examination of structure matrix correlations reveals that K_2_O is the most important predictor of region without controlling for the remaining predictors, with function 1 extracting 73% of the variance from K_2_O. Examination of standardized function coefficients indicates that the most important predictors in function 1 after controlling for the remaining predictors (i.e., the predictors making the largest unique contributions) are K_2_O (1.08) and SiO_2_ (− 0.78).

**Table 3:** Structure matrix correlations and standardized function coefficients from discriminant function analysis performed using the weight percentages (centered log-ratio transformed) of ten oxides as predictors of region (Cape Verde Islands, Mid-African Rift, East African Rift, Red Sea Rift, and Hawaiian Islands; *n* = 10,553). The largest structure matrix correlation for a given oxide is starred

Oxide	Structure matrix correlations				Standardized function coefficients			
	1	2	3	4	1	2	3	4
K_2_O	0.85^*^	0.05	− 0.06	0.38	1.08	0.43	0.03	0.17
CaO	− 0.61^*^	0.16	0.25	− 0.31	0.28	0.04	− 0.09	− 0.79
MgO	− 0.58^*^	0.19	0.32	− 0.42	− 0.05	0.40	1.44	− 0.07
Na_2_O	0.54^*^	− 0.38	0.12	0.37	− 0.17	0.03	0.95	− 0.44
Fe_2_O_3_	− 0.46^*^	− 0.42	− 0.16	0.29	0.21	− 0.81	− 0.23	0.43
SiO_2_	0.04	− 0.72^*^	− 0.04	0.32	− 0.78	0.35	0.13	0.17
Al_2_O_3_	0.17	− 0.68^*^	0.20	0.43	0.29	− 0.69	0.57	0.75
P_2_O_5_	0.11	0.64^*^	0.36	0.17	0.04	0.04	0.56	0.47
TiO_2_	− 0.55	0.60^*^	0.01	0.39	− 0.66	0.85	− 0.06	0.70
MnO	0.17	− 0.43^*^	− 0.28	0.22	0.13	0.39	− 0.37	− 0.11

Function 2 (*Λ* = 0.75, *χ*^2^(27, *n* = 10,553) = 3021.48, *p* < 0.001, *η*^2^ = 0.19) has 19% of its variance explained by region and accounts for 21% of the model’s between-groups variance. Structure matrix correlations reveal the most important predictor is SiO_2_, with function 2 extracting 52% of the variance from SiO_2_. The predictors making the largest unique contributions to function 2 are TiO_2_ (0.85) and Fe_2_O_3total_ (− 0.81). Function 3 (*Λ* = 0.93, *χ*^2^(16, *n* = 10,553) = 787.33, *p* < 0.001, *η*^2^ = 0.04) has 4% of its variance explained by region and accounts for 4% of the model’s between-groups variance. Large unique contributions to function 3 come from MgO (1.44), Na_2_O (0.95) and P_2_O_5_ (0.56). Function 4 (*Λ* = 0.97, *χ*^2^(7, *n* = 10,553) = 340.66, *p* < 0.001, *η*^2^ = 0.03) has 3% of its variance explained by region and accounts for 3% of the model’s between-groups variance, with large unique contributions from Al_2_O_3_ (0.75) and CaO (− 0.79).

Classification results (Table 4) reveal that 78.4% of all cases are identified correctly (compared to an expected hit ratio by chance of 54.4%). Cross-validation results (data not shown) are virtually identical, with 78.4% cases identified correctly. In order to assess the accuracy of prediction of group membership while taking into account chance agreement, we computed a kappa coefficient (Green and Salkind 2005) and obtained a value of 0.48 indicating moderate accuracy in prediction. Hawaiian Islands and the East African Rift have the highest levels of correct classification (92.4 and 53.5%, respectively). Cape Verde Islands, the Mid-African Rift, and Red Sea Rift are more frequently misclassified as the Hawaiian Islands (57.4, 51.2, and 46.8%, respectively) than correctly classified. The Mid-African Rift is also frequently misclassified as East African Rift (24.4%).

**Table 4:** Original classification results from discriminant function analysis performed using the weight percentages (centered log-ratio transformed) of ten oxides as predictors of region (CV = Cape Verde Islands, MAR = Mid-African Rift, EAR = East African Rift, RS = Red Sea Rift, HI = Hawaiian Islands; *n* = 10,553). Results reveal 78.4% of all cases identified correctly (expected hit ratio by chance of 54.4%)

	CV	MAR	EAR	RS	HI	Total
Count						
CV	247	1	58	0	412	718
MAR	39	38	80	3	168	328
EAR	133	48	1030	33	682	1926
RS	3	1	8	30	37	79
HI	57	57	369	86	6933	7502
%						
CV	34.4	0.1	8.1	0.0	*57.4*	100
MAR	11.9	11.6	24.4	0.9	*51.2*	100
EAR	6.9	2.5	*53.5*	1.7	35.4	100
RS	3.8	1.3	10.1	38.0	*46.8*	100
HI	0.8	0.8	4.9	1.1	*92.4*	100

Italicized location that received highest percent classification

Note that all African regions, except the East African Rift, are misclassified as the Hawaiian Islands

**Fig. 9:**
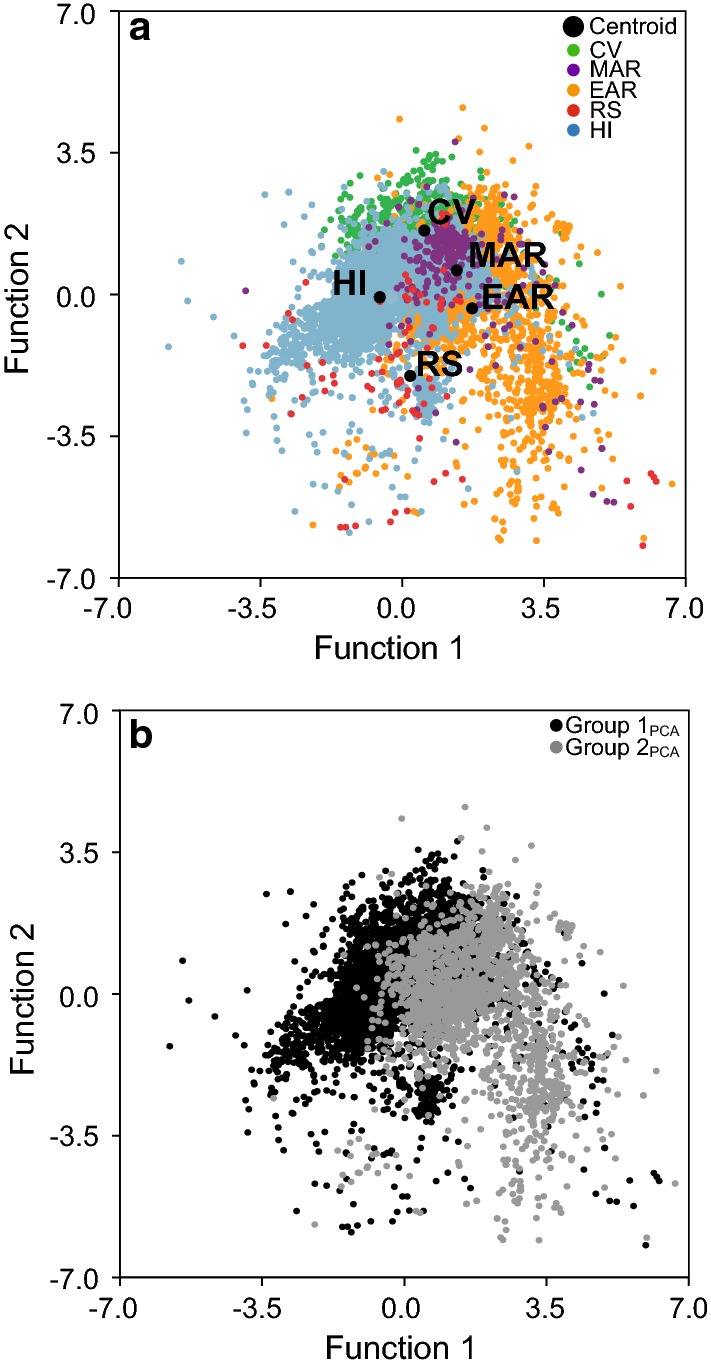
Discriminant function scores on functions 1 and 2 (centered log-ratio transformed data [*n* = 10,553]) color coded to show **a** five regions (CV = Cape Verde Islands, MAR = Mid-African Rift, EAR = East African Rift, RS = Red Sea Rift, HI = Hawaiian Islands) and **b** Group 1_PCA_ and Group 2_PCA_

The mean discriminant score for each region by discriminant function is presented in Table 5, and a plot depicting individual cases and region group centroids in discriminant function space defined by the first two discriminant functions is shown in Fig. 9a. The same discriminant function scores plot as in Fig. 9a was coded by group classification defined by PCA to produce Fig. 9b. An independent samples *t*-test determines that scores on discrimination function 1 are larger for Group 2_PCA_ (3.13 ± 0.03) than for Group 1_PCA_ (− 0.22 ± 0.01; *t*(871.17) = −98.58, *p* < 0.001, Cohen’s *d* = −3.15). In contrast, scores on discriminant function 2 are larger for Group 1_PCA_ (0.11 ± 0.01) than for Group 2_PCA_ (− 1.56 ± 0.07; *t*(734.34) = 25.32, *p* < 0.001, Cohen’s *d* = 1.62).

**Table 5:** Mean discriminant score for each region by discriminant function from discriminant function analysis performed using the weight percentages (centered log-ratio transformed) of ten oxides as predictors of region (CV = Cape Verde Islands, MAR = Mid-African Rift, EAR = East African Rift, RS = Red Sea Rift, HI = Hawaiian Islands; *n* = 10,553)

	DF 1	DF 2	DF 3	DF 4
CV	0.54	1.58	− 0.05	− 0.30
MAR	1.34	0.60	0.91	0.53
EAR	1.73	− 0.34	− 0.14	0.00
RS	0.19	− 2.01	1.36	− 1.54
HI	− 0.56	− 0.07	− 0.01	0.02

Data from PCA-coded groups (Fig. 9b), in conjunction with structure matrix correlations (Table 3), reveal that function 1 discriminates well between Group 1_PCA_ (characterized by smaller relative amounts of K_2_O) and Group 2_PCA_ (characterized by larger relative amounts of K_2_O). Conceptualized in terms of locations (Fig. 9a), function 1 shows the largest separation between the Hawaiian Islands and the East African Rift (confirmed by calculating the difference in mean discriminant scores on DF1 from Table 5), though there is considerable overlap between the African locations. In comparison with function 1, function 2 shows relatively minimal discrimination between Group 1_PCA_ (characterized by smaller relative amounts of SiO_2_ and Al_2_O_3_) and Group 2_PCA_ (characterized by larger relative amounts of SiO_2_ and Al_2_O_3_) and provides no discrimination between landmasses (i.e., African vs. Hawaiian cases).

Considering discriminant function scores on all four functions simultaneously, an examination of pairwise group comparisons (Table 6) indicates that every region’s centroid is significantly distant from every other region’s centroid (*p* < 0.001 for all comparisons). The *F* statistics from pairwise group comparisons (the magnitudes of which can be used to compare Mahalanobis distances between groups in multivariate space) reveal the largest separation exists between the Hawaiian Island and East African Rift centroids (*F* = 810.73), while the smallest separation is between East African Rift and Mid-African Rift (*F* = 67.10).

**Table 6:** Pairwise comparisons of distance between group centroids in discriminant function space defined by all four discriminant functions for the five regions (CV = Cape Verde Islands, MAR = Mid-African Rift, EAR = East African Rift, RS = Red Sea Rift, HI = Hawaiian Islands; *n* = 10,553)

	EAR	HI	MAR	RS
CV				
*F*	271.08	263.82	72.23	117.82
*p*	< 0.001	< 0.001	< 0.001	< 0.001
EAR				
*F*		810.73	67.10	74.00
*p*		< 0.001	< 0.001	< 0.001
HI				
*F*			161.90	67.76
*p*			< 0.001	< 0.001
MAR				
*F*				80.24
*p*				< 0.001

The largest separation is between the Hawaiian Island and East African Rift centroids

### Analysis of silicon:aluminum ratio by region

Price and Henderson (1978) hypothesized that podoconiosis may be associated with the ratio of silicon to aluminum in the soil. By testing for a difference in the SiO_2_:Al_2_O_3_ ratio among the five regions, including those associated with (Cape Verde Islands, Mid-African Rift, East African Rift, Red Sea Rift) and those not associated with (Hawaii) podoconiosis, we sought to explore this hypothesis. Thus, we conducted a one-way ANOVA treating the ratio of clr-transformed SiO_2_ to clr-transformed Al_2_O_3_ as the dependent variable and region as a between-subjects factor. Ratios less than 1 indicate that, on average, the deviation from the geometric mean of the composition for Al_2_O_3_ is greater than that of SiO_2_. Results indicate a significant difference in the SiO_2_:Al_2_O_3_ ratio among regions (*F*(4, 10,548) = 211.28, *p *< 0.001, *η*^2^ = 0.07), with 7% of the variance in the ratio explained by region. Tamhane post hoc tests reveal the ratio for the Cape Verde Islands (0.50 ± 0.002) is significantly lower than for all other regions (Cohen’s *d* 0.58–0.94). Furthermore, the ratio for the Hawaiian Islands (0.53 ± 0.001), while significantly greater than that of Cape Verde Islands (Cohen’s *d* 0.58), is significantly lower than for all remaining regions (Cohen’s *d* 0.55–0.98). The SiO_2_:Al_2_O_3_ ratio of the East African Rift (0.55 ± 0.001), Mid-African Rift (0.56 ± 0.003), and the Red Sea Rift (0.57 ± 0.005) is not significantly different from one another (Cohen’s *d* 0.10–0.26). A nonparametric Kruskal–Wallis ANOVA (results not shown) yields results identical to the parametric ANOVA. Untransformed SiO_2_:Al_2_O_3_ ratios can be found in Online Resource 3.

**Fig. 10:**
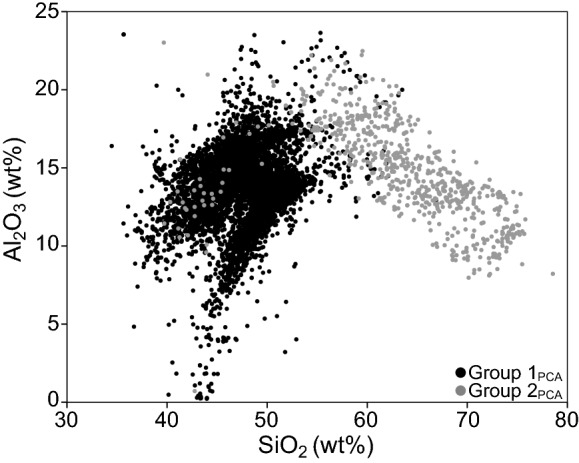
Harker diagram of SiO_2_ vs. Al_2_O_3_ (*n* = 10,553) for Group 1_PCA_ and Group 2_PCA_ using **a** untransformed weight percent and **b** centered log-ratio transformed weight percent

In addition to comparing SiO_2_:Al_2_O_3_ ratios among regions, we also wanted to determine whether there is a significant difference in this ratio between Group 1_PCA_ (*n *= 9849) and Group 2_PCA_ (*n *= 704). A pattern in the Harker diagram (bivariate scatterplot of SiO_2_ vs. Al_2_O_3_; Fig. 10) shows Group 2_PCA_ further to the right (higher SiO_2_) for a given weight% Al_2_O_3_ than Group 1_PCA_ cases. An independent samples *t* test reveals the untransformed ratio for Group 2_PCA_ (0.58 ± 0.002) is significantly larger than for Group 1_PCA_ (0.53 ± 0.001; *t*(785.23) = −26.09, *p* < 0.001, Cohen’s *d* = 1.12).

## Discussion

Podoconiosis is a complicated disease to study, not only because of the complexity of the human body, but also because of the disease’s large and varied geographic range. The current model of podoconiosis posits fine-grained minerals entering the lymphatic system leading to an inflammatory response. However, within Ethiopia, for example, the presence of the disease in only certain towns suggests other causative factors could be a local soil composition or texture that should be related to the bedrock composition. Indeed, research into podoconiosis has consistently noted the local variability of the disease and its associated soils (Oomen 1969; Price 1974a, b; Price et al. 1981; Deribe et al. 2013, 2015). Previous investigation of podoconiosis correlated the disease with the “tropical red soil” and, by extension, its source rock (Price et al. 1981; Price and Bailey 1984). This current, exploratory study searches for specific bedrock elements or element suites associated with the disease with an aim to narrow future geological research efforts. Results of this study support previous hypotheses that a unique alkaline- and silica-rich geochemistry is associated with podoconiosis and provide suggestions for future research.

We propose there is a unique geochemistry associated with Group 2 that is of interest to podoconiosis research. The presence of a unique geochemical subset worthy of deeper analysis was first identified in the two-cluster separation of cases that is readily apparent by AFM (Fig. 2), TAS (Fig. 3), principal component scores diagram (Fig. 5), and discriminant function scores diagram (Fig. 9). Although the findings from these plots and analyses are not independent given that they share sets or subsets of variables, the fact that the same cases repeatedly show up in the same cluster (i.e., either Group 1 or Group 2) among analyses (see Fig. 7) lends support for the presence of a unique geochemistry of Group 2. The presence of East African Rift cases in both Group 1 and Group 2 is consistent with literature on podoconiosis which reports that the incidence of the disease in Ethiopia is locally variable. The lack of clearly defined geographic borders between Group 1_PCA_ and Group 2_PCA_ (Fig. 6) mimics, in a general sense, the local-scale variation in the presence/absence of podoconiosis. By coding our cases into Group 1 or Group 2 based on which side of the line of separation they fell (Figs. 2, 3, 5), we were able to identify which elements contribute most to the separation of these two groups. The highly alkaline nature of Group 2 geochemistry is supported by both principal component 1 and discriminant function 1, which are most associated with Mg, Ca, Na, and K. In addition to confirming high alkalinity, our TAS diagram also supports a high silica content for Group 2. This is confirmed by principal component 2 and discriminant function 2, which are associated with Si and Al. This particular geochemical combination, high alkalinity and high silica content, is atypical among volcanic compositions. Alkaline igneous rocks make up only about 1% of all rocks worldwide, and of these, most are silica-depleted (Fitton and Upton 1987). This unique geochemistry of bedrock implies a unique suite of minerals which, with time and local weathering conditions, will lead to a unique soil. We propose that the composition of bedrock identified by Group 2, when weathered, leads to the production of elemental subcomponents that may play a role in the onset of podoconiosis.

Principal component 1 and discriminant function 1: Enrichment in incompatible elements is unique to Group 2 and may provide a significant geochemical clue for determining the nature of the “irritant particles.”

The separation of cases along principal component 1 in Fig. 5 and along discriminant function 1 in Fig. 9 is associated with relative abundances of Mg, Ca, Na, and K. In standard igneous petrology, the terms compatible and incompatible describe the behavior of elements within the magma melt system and their affinity for staying in the solid or concentrating into the liquid magma, respectively. This process controls both major and trace element distribution in the rocks and is indicative of the amount of magma differentiation. Relative amounts of Mg, Ca, Na, and K are commonly used to characterize a highly evolved magma from its more primitive progenitor (Ragland 1989; Rollinson 1993). Mg and Ca are compatible, whereas K and Na are incompatible. Interpretation of PCA and DFA results indicates that Group 2 cases are characterized by greater relative abundances of incompatible elements compared to Group 1 cases. Because principal component 1 has large positive loadings from Mg and Ca and large negative loadings from K and Na (Fig. 4), the relatively lower scores of Group 2 on component 1 (Fig. 5) indicate lower relative abundances of Mg and Ca and higher relative abundances of K and Na in comparison with Group 1. Likewise, because discriminant function 1 has substantial negative structure matrix correlations with Mg and Ca and substantial positive correlations with K and Na (Table 3), the relatively higher scores of Group 2 on function 1 (Fig. 9) also indicate lower relative abundances of Mg and Ca and higher relative abundances of K and Na in comparison with Group 1. This link with incompatible elements is supported by the results of local soil research by Le Blond et al. (2017) who also report an increase in K and Na in endemic soils.

Alternatively, the cause of podoconiosis may not be enrichment in major incompatible elements (or their associated minerals), but rather trace elements associated with them. Some podoconiosis literature has mentioned the presence of trace elements, specifically Be, Ce, Co, Cr, Cu, La, Nd, Ni, S, Sn, V, Zn, and Zr (Price and Pitwell 1973; Price and Henderson 1978; Frommel et al. 1993). Frommel and his colleagues argue strongly that Be and Zr are of particular interest due to the elevated levels of these elements they found in soils associated with podoconiosis, along with the ability of both to form granular masses within the body. Certain trace elements follow similar substitution patterns to those of the major elements within melt systems. For instance, the large ion lithophile (LIL) elements Rb and Cs can substitute for the elements Na and K, the incompatible elements identified as important in Group 2 (Rollinson 1993). In contrast, Frommel et al. (1993) reported elevated levels of Ba and Sr in soils from towns where podoconiosis is not present. This supports our hypothesis as these trace elements could follow the trends of compatible elements (Rollinson 1993). We hypothesize that specific trace elements predicted by the presence of incompatible major elements may play a role in the etiology of podoconiosis. Recent soil research has reported higher levels of Cr, Ni, Si, Y, and Zr within endemic soils (Molla et al. 2014; Le Blond et al. 2017). Future work that focuses on tracking the pathway of the trace elements enriched in Group 2 to the podoconiosis-associated soils may be key to the etiology of podoconiosis.

Principal component 2 and discriminant function 2: Silica and alumina are responsible for minimal separation between Group 1 and Group 2, and their role in podoconiosis may be less significant than previous literature suggests.

Silica is often named alone, or in combination with aluminum, as the “irritant particle” in podoconiosis literature (Heather and Price 1972; Price and Pitwell 1973; Price and Henderson 1979; Price and Bailey 1984; Fyfe and Price 1985; Price 1988; Frommel et al. 1993). Our analyses reveal principal component 2 and discriminant function 2 are associated with Si and Al (Fig. 4, Table 3). The geochemical behavior of Si and Al is slightly different than that of the compatible/incompatible elements due to their high abundance in the crust. Higher relative amounts of Si and Al are frequently correlated with magma at relatively shallow levels in the crust (Rollinson 1993; Best and Christiansen 2001). In addition to the major role that rifting plays in the African regions, plume effects are also present to varying degrees (Déruelle et al. 1991; Anderson and Schramm 2005; Furman et al. 2006). Since plumes originate from deeper sources, their magma contains relatively low amounts of silica. Thus, the greater the effect of rift volcanics, the higher the silica; the greater the effect of plume volcanics, the lower the silica. Results from our AFM, TAS, PCA, and DFA (Figs. 2a, 3a, 5a, 9a) frequently found the Cape Verde Islands and the Red Sea Rift showing more similar geochemistry to that of the Hawaiian Islands than we expected. We attribute this similarity to the higher proportion of plume volcanics associated with their formation than of the other regions (Anderson and Schramm 2005; Furman et al. 2006; Ramalho 2011).

A comment needs to be made about the unexpected loading of Mn near Si and Al on principal component 2 as Mn is a compatible element and normally follows the same magmatic evolution patterns to that of Mg or Fe. We believe the observed loading is influenced by Hawaiian cases and follows from the inclusion of dacite melts with picritic melts in the Hawaiian lavas as suggested by Huang et al. (2007).

Some differences in the relative abundances of Si and Al among the five regions can be estimated upon visual inspection of both the principal component scores and discriminant function scores plots (Figs. 5a, 9a), as component/function 2 are associated with Si and Al. The Cape Verde Islands and Mid-African Rift tend to plot lower on component 2 and higher on function 2, indicating relatively lower silica and aluminum, than the East African Rift and the Hawaiian Islands. However, a subtler separation along component/function 2 is visible when comparing plots coded by Group. Though Groups 1 and 2 show similar ranges along both component 2 and function 2, mean values for the Groups are different on both. These results indicate a relatively higher silicon and aluminum content for Group 2, whose geochemistry we propose is associated with podoconiosis. However, Cohen’s *d* for the comparison of Group 1 and 2 on both component 2 and function 2 is approximately half that for component 1 and function 1, suggesting that the importance of component/function 2, and thus Si and Al, is relatively minimal. Le Blond et al. (2017) report a significant difference in mean values for both Si and Al between endemic and nonendemic soils. This difference between bedrock and soil geochemistry might suggest enrichment due to weathering or anthropological activities.

Another line of research has suggested that it is not merely the presence of silicon and/or aluminum in soils, but their ratio that is of importance to podoconiosis. Several studies (Heather and Price 1972; Price and Henderson 1978, 1979; Price et al. 1981; Blundell et al. 1989) analyzed particles found in the lymph nodes of individuals affected by and not affected by podoconiosis. These studies found no difference in the number of particles between affected and unaffected individuals. However, a statistically significant difference in the silicon:aluminum ratio was reported and suggested to be of importance to the disease process. As a preliminary investigation of this hypothesis, we tested for a difference in the SiO_2_:Al_2_O_3_ ratio among the five regions with no clear results. The Mid-African Rift, East African Rift, and Red Sea Rift do have higher mean ratios than does the Hawaiian Islands. However, the Cape Verde Islands mean is lower than that of Hawaii, so there is no clear separation of the podoconiosis-associated regions from non-associated region. When considered in terms of Groups, we found Group 2 had a significantly larger SiO_2_:Al_2_O_3_ ratio than that of Group 1, suggesting a higher ratio in areas we propose is associated with podoconiosis. However, the ubiquitous nature of silicon and aluminum, when considered both independently or in association with each other, around the world with no widespread prevalence of podoconiosis (despite the lack of shoes in many countries) is by far the greatest challenge to this theory based on composition alone.

## Electronic supplementary material

Below is the link to the electronic supplementary material.

Supplementary material 1 (DOCX 24 kb)

Supplementary material 2 (EPS 30803 kb)

Supplementary material 3 (DOCX 12 kb)
