# Supplementary material for: Regional bedrock geochemistry associated with podoconiosis evaluated by multivariate analysis
Source: Environ Geochem Health. 2018 Sep 5;41(2):649–65. doi: 10.1007/s10653-018-0158-0 (PMC6510837; doi:10.1007/s10653-018-0158-0)
Supplement: Supplementary file 1 — Supplementary material 1 (DOCX 24 kb) [file 10653_2018_158_MOESM1_ESM.docx]

**Online Resource 1** Descriptive statistics of normalized untransformed weight percentages of 10 oxides from each of the six regions studied (CL=Cameroon Line, CV=Cape Verde Islands, MAR=Mid-African Rift, EAR=East African Rift, RS=Red Sea Rift, HI=Hawaiian Islands). CoV denotes coefficient of variation

|  | *n* | Mean | SD | CoV | Min | Max |  | *n* | Mean | SD | CoV | Min | Max |
| --- | --- | --- | --- | --- | --- | --- | --- | --- | --- | --- | --- | --- | --- |
| **SiO_2_** |  |  |  |  |  |  | **Al_2_O_3_** |  |  |  |  |  |  |
| CL | 44 | 50.36 | 6.31 | 12.53 | 39.89 | 63.96 | CL | 44 | 15.96 | 5.15 | 32.27 | 7.25 | 24.58 |
| CV | 762 | 44.28 | 3.81 | 8.60 | 35.78 | 64.04 | CV | 762 | 13.26 | 3.56 | 26.85 | 0.22 | 23.72 |
| MAR | 406 | 52.61 | 9.81 | 18.65 | 38.06 | 79.78 | MAR | 406 | 15.26 | 2.59 | 16.97 | 0.45 | 23.46 |
| EAR | 2346 | 55.22 | 10.77 | 19.50 | 35.16 | 88.00 | EAR | 2346 | 14.06 | 3.69 | 26.24 | 0.09 | 38.17 |
| RS | 124 | 49.76 | 5.19 | 10.43 | 40.87 | 71.32 | RS | 124 | 12.76 | 5.88 | 46.08 | 1.45 | 21.93 |
| HI | 8319 | 50.25 | 2.68 | 5.33 | 36.61 | 76.60 | HI | 8319 | 13.61 | 1.87 | 13.74 | 0.47 | 23.62 |
| **MgO** |  |  |  |  |  |  | **Fe_2_O_3_** |  |  |  |  |  |  |
| CL | 44 | 6.52 | 5.98 | 91.72 | 0.03 | 18.74 | CL | 44 | 8.80 | 4.82 | 54.77 | 1.56 | 15.04 |
| CV | 762 | 10.83 | 7.67 | 70.82 | 0.13 | 47.75 | CV | 762 | 11.43 | 4.82 | 42.17 | 1.56 | 15.04 |
| MAR | 406 | 5.75 | 5.08 | 88.35 | 0.00 | 48.44 | MAR | 406 | 9.11 | 3.61 | 39.63 | 0.96 | 16.94 |
| EAR | 2335 | 5.87 | 8.09 | 137.82 | 0.00 | 51.94 | EAR | 2346 | 9.05 | 3.40 | 37.57 | 0.13 | 19.26 |
| RS | 124 | 15.99 | 14.26 | 89.18 | 0.01 | 44.14 | RS | 124 | 8.17 | 2.06 | 25.21 | 2.21 | 13.43 |
| HI | 8319 | 8.26 | 4.28 | 51.82 | 0.03 | 46.21 | HI | 8319 | 11.39 | 1.26 | 11.06 | 0.77 | 19.92 |
| **TiO_2_** |  |  |  |  |  |  | **MnO** |  |  |  |  |  |  |
| CL | 42 | 1.93 | 1.24 | 64.25 | 0.05 | 3.80 | CL | 36 | 0.15 | 0.09 | 60.00 | 0.01 | 0.37 |
| CV | 762 | 3.42 | 1.12 | 32.75 | 0.01 | 6.48 | CV | 762 | 0.19 | 0.03 | 15.79 | 0.01 | 0.41 |
| MAR | 406 | 2.29 | 1.34 | 58.52 | 0.00 | 8.75 | MAR | 405 | 0.18 | 0.08 | 44.44 | 0.00 | 0.74 |
| EAR | 2346 | 1.70 | 1.18 | 69.41 | 0.00 | 8.45 | EAR | 2336 | 0.20 | 0.23 | 115.00 | 0.00 | 13.34 |
| RS | 124 | 1.13 | 0.87 | 76.99 | 0.01 | 2.96 | RS | 124 | 0.17 | 0.15 | 88.24 | 0.04 | 1.23 |
| HI | 8316 | 2.54 | 0.67 | 26.38 | 0.00 | 6.86 | HI | 7658 | 0.20 | 0.43 | 215.00 | 0.00 | 13.77 |
| **CaO** |  |  |  |  |  |  | **Na_2_O** |  |  |  |  |  |  |
| CL | 44 | 7.02 | 4.22 | 60.11 | 0.66 | 13.39 | CL | 44 | 5.69 | 3.33 | 58.52 | 1.51 | 13.10 |
| CV | 762 | 11.29 | 3.29 | 29.14 | 0.17 | 23.52 | CV | 762 | 3.18 | 1.73  73 | 54.40 | 0.00 | 11.59 |
| MAR | 406 | 7.66 | 4.44 | 57.96 | 0.00 | 20.35 | MAR | 406 | 4.19 | 1.34 | 31.98 | 0.11 | 8.90 |
| EAR | 2345 | 6.76 | 4.80 | 71.01 | 0.00 | 26.12 | EAR | 2346 | 4.18 | 1.84 | 44.02 | 0.02 | 14.52 |
| RS | 124 | 8.49 | 4.35 | 51.24 | 0.70 | 15.82 | RS | 124 | 2.71 | 1.94 | 71.59 | 0.00 | 10.64 |
| HI | 8319 | 10.29 | 1.68 | 16.33 | 0.05 | 17.39 | HI | 8318 | 2.55 | 0.87 | 34.12 | 0.01 | 9.76 |
| **K_2_O** |  |  |  |  |  |  | **P_2_O_5_** |  |  |  |  |  |  |
| CL | 44 | 3.18 | 2.03 | 63.84 | 0.79 | 7.56 | CL | 41 | 0.54 | 0.39 | 72.22 | 0.08 | 1.44 |
| CV | 762 | 1.43 | 1.21 | 84.62 | 0.00 | 8.49 | CV | 762 | 0.69 | 0.33 | 47.83 | 0.00 | 2.54 |
| MAR | 400 | 2.45 | 1.61 | 65.71 | 0.00 | 6.98 | MAR | 390 | 0.55 | 0.34 | 61.82 | 0.00 | 1.75 |
| EAR | 2319 | 2.64 | 1.93 | 73.11 | 0.00 | 18.51 | EAR | 2239 | 0.40 | 0.38 | 95.00 | 0.00 | 5.60 |
| RS | 124 | 0.63 | 1.15 | 182.54 | 0.00 | 5.57 | RS | 112 | 0.21 | 0.24 | 114.29 | 0.00 | 1.19 |
| HI | 8296 | 0.61 | 0.52 | 85.25 | 0.00 | 6.05 | HI | 8164 | 0.33 | 0.22 | 66.67 | 0.00 | 3.01 |
