# Supplementary material for: Regional bedrock geochemistry associated with podoconiosis evaluated by multivariate analysis
Source: Environ Geochem Health. 2018 Sep 5;41(2):649–65. doi: 10.1007/s10653-018-0158-0 (PMC6510837; doi:10.1007/s10653-018-0158-0)
Supplement: Supplementary file 3 — Supplementary material 3 (DOCX 12 kb) [file 10653_2018_158_MOESM3_ESM.docx]

**Online Resource 3** Mean ± 1 SD SiO_2_:Al_2_O_3_ ratio (untransformed) for each of the five regions (CV=Cape Verde Islands, MAR=Mid-African Rift, EAR=East African Rift, RS=Red Sea Rift, HI=Hawaiian Islands; *n* = 10,553)

|  | Mean | SD |
| --- | --- | --- |
| CV | 0.30 | 0.002 |
| MAR | 0.32 | 0.002 |
| EAR | 0.29 | 0.002 |
| RS | 0.29 | 0.010 |
| HI | 0.27 | 0.0004 |
